# M6A Classification Combined With Tumor Microenvironment Immune Characteristics Analysis of Bladder Cancer

**DOI:** 10.3389/fonc.2021.714267

**Published:** 2021-09-15

**Authors:** Huili Zhu, Xiaocan Jia, Yuping Wang, Zhijuan Song, Nana Wang, Yongli Yang, Xuezhong Shi

**Affiliations:** Department of Epidemiology and Biostatistics, College of Public Health, Zhengzhou University, Zhengzhou, China

**Keywords:** m6A, bladder cancer, mutation burden, tumor microenvironment, immunotherapy

## Abstract

**Background:**

Studies have shown that N6-methyl adenosine (m6A) plays an important role in cancer progression; however, the underlying mechanism of m6A modification in tumor microenvironment (TME) cell infiltration of bladder cancer remains unclear. This study aimed to investigate the role of m6A modification in TME cell infiltration of bladder cancer.

**Methods:**

The RNA expression profile and clinical data of bladder cancer were obtained from The Cancer Genome Atlas and Gene Expression Omnibus. We assessed the m6A modification patterns of 664 bladder cancer samples based on 20 m6A regulators through unsupervised clustering analysis and systematically linked m6A modification patterns to TME cell infiltration characteristics. Gene ontology and gene set variation analyses were conducted to analyze the underlying mechanism based on the assessment of m6A methylation regulators. Principal component analysis was used to construct the m6A score to quantify m6A modification patterns of bladder cancer.

**Results:**

The genetic and expression alterations in m6A regulators were highly heterogeneous between normal and bladder tissues. Three m6A modification patterns were identified. The cell infiltration characteristics were highly consistent with the three immune phenotypes, including immune rejection, immune inflammation, and immune desert. The biological functions of three m6A modification patterns were different. Cox regression analyses revealed that the m6A score was an independent signature with patient prognosis (HR = 1.198, 95% CI: 1.031–1.390). Patients with a low-m6A score were characterized by increased tumor mutation burden, PD-L1 expression, and poorer survival. Patients in the low-m6A score group also showed significant immune responses and clinical benefits in the CTLA-4 immunotherapy cohort (*p* =0.0069).

**Conclusions:**

The m6A methylation modification was related to the formation of TME heterogeneity and complexity. Assessing the m6A modification pattern of individual bladder cancer will improve the understanding of TME infiltration characteristics.

## Introduction

Post-transcriptional modification is an important regulatory step in many physiological and disease progressions. More than 100 different types of post-transcriptional RNA chemical modifications have been identified in organisms (1). N6-methyl adenosine (m6A), one of the most abundant modifications in eukaryotic cells, has been identified as a post-transcriptional regulatory factor in various types of RNA, including messenger RNA, microRNA, and long non-coding RNA. It is also considered to be the most common RNA molecule with abundant modifications and plays an important role in the development of tumors (2). Like DNA and protein modification, m6A modification is a reversible process regulated by writers, readers, and erasers (3). Although the m6A methylation immunoprecipitation high-throughput sequencing technology has broken the understanding of m6A methylation site modification, the RNA fragments targeted by the technology are limited to around 100 nt long; thus, the methylation sites altered by single nucleotides cannot be detected (4). While the photo cross-linking assists m6A sequencing technology and m6A single-base resolution, purple foreign precipitation technology made the RNA m6A methylation site detection more accurate (5). In addition, the m6A regulatory factor is closely related to the activity of the urinary system tumor-related signaling pathways (6); therefore, exploring the relationship between m6A regulatory molecules and target gene RNA modification will help in understanding the mechanism behind the occurrence and development of bladder cancer.

The tumor microenvironment (TME) can promote tumor cell proliferation, invasion, and metastasis by regulating different signaling pathways (7). In the TME, certain types of lymphocytes can infiltrate into the tumor interior, which are called tumor-infiltrating lymphocytes, including T lymphocytes, B lymphocytes, and antigen-presenting dendritic cells (8, 9). Tumor-infiltrating lymphocytes mediate immunosuppression of the TME, which can help tumor cells achieve immune escape and then promote malignant development of tumors (10); therefore, different tumor immunophenotypes may be identified by analyzing the complexity and heterogeneity of the TME. The accurate prediction of the clinical efficacy of different immunotherapeutic approaches would also be improved (11, 12).

Recent studies have shown that different m6A modifications play an important role in different biological processes, such as inflammation, innate immunity, and TME (13–16). It has been shown that methylation of mRNA m6A accelerated the activation and function of dendritic cells (17). Li et al. (18) found that m6A-modified methylation controlled the steady-state differentiation of T cells by controlling the IL-7/STAT5/SOCS signaling pathway. Due to technical limitations, these studies were necessarily limited to one or two m6A regulators and cell types, but the antitumor effect was characterized by multiple tumor suppressor factors interacting through a high degree of synergy. The potential role of m6A modification in the tumor TME cell infiltration of bladder cancer has not been reported; hence, this study aimed to elucidate the role of m6A methylation modification combined with the TME of bladder cancer.

## Material and Methods

### Bladder Cancer Data Sources and Study Design

TCGA-BLCA (a dataset that included RNA sequencing data, genome mutation data, and clinical data) was downloaded from The Cancer Genome Atlas (TCGA) (https://tcga-data.nci.nih.gov/tcga/, accessed on January 12, 2020) (19). GSE13507 (a dataset that included RNA sequencing data and clinical data) was downloaded from the Gene-Expression Omnibus (GEO) (https://www.ncbi.nlm.nih.gov/geo/, accessed on January 12, 2020) (20). The transcripts per kilobase million (TPM) value was closer to the data of the GEO chip. We used the fpkm function of the “limma” package in R to convert the FPKM value of the RNA data to the TPM value (21). Compliant data sets were subjected to copy number variation (CNV) analysis. The plot of m6A regulator copy number changes in the chromosome was drawn using the “Rcircos” package.

### NMF Consensus Molecular Clustering of 20 m6A Modulators

We used 20 m6A regulators to determine different m6A methylation modifications in bladder cancer, including 12 readers (YTHDC1, HNRNPA2B1, YTHDC2, FMR1, YTHDF1, YTHDF2, YTHDF3, IGF2BP1, IGF2BP2, IGF2BP3, LRPPRC, RBMX), 7 writers (METTL3, ZC3H13, METL16, RBM15, RBM15B, WTAP, VIRMA) and 1 eraser (ALKBH5). According to the expression of 20 m6A regulators, unsupervised cluster analysis in the “ConsensuClusterPlus” package was used to identify different m6A modification patterns.

### Gene Set Variation Analysis and Gene Enrichment Function Annotation

We downloaded the gene sets of the “c2.cp.kegg.v6.2 symbol” from the Molecular Signatures Database (MSigDB) (22). Then, the “GSVA” package for enrichment analysis was used to study the difference in the activities of m6A modification patterns in biological processes (23). The gene ontology (GO) function annotations of m6A-modified phenotype-related genes were analyzed using the “clusterProfiler” package and FDR <0.01.

### Immune Cell Difference Analysis

The TME-infiltrating immune cell gene set was obtained from the research of Pornpimol Charoentong. The gene set had a variety of human immune cell subtypes, including activated CD8 T cells, activated dendritic cells, giant natural killer T cells, and regulatory T cells. The single sample gene set enrichment analysis (ssGSEA) algorithm quantified the immune cell infiltration in bladder cancer TME. The difference analysis of immune cells was used to observe the difference between the m6A patterns of immune cells.

### Screening of Differentially Expressed Genes Among Different Phenotypes of m6A

Different m6A modification patterns were typed by the consensus clustering algorithm. The R package “limma” screened the m6A differentially expressed genes (DEG) between different m6A phenotypes. The gene with adjusted *p* < 0.001 was deemed as significant DEG. The relationship between m6A gene characteristics and related biological pathways was further explored through the correlation analysis.

### Construction of m6A Gene Signature

Differential genes determined in different m6A clusters were normalized in bladder cancer samples to extract crossover genes. The unsupervised clustering method was used to analyze the degree of overlap, with the patients divided into several groups for further analysis. The consensus clustering algorithm was used to determine the number of gene clusters and their stability. Then, univariate Cox regression analysis was used to analyze the prognosis of each gene. Taking into account the correlation between genes, the traditional Cox regression model was not used directly; therefore, the differential genes related to prognosis obtained by univariate Cox regression were further analyzed with principal component analysis (PCA). Finally, PCA analysis was applied to construct the m6A-related gene signature and evaluate the m6A gene signature of each bladder cancer patient, which was called m6A score. Patients were divided into the high-score group and low-score group based on the maximally selected rank statistics.

### Statistical Analysis

Correlation coefficients between the TME-infiltrating immune cells and the expression of m6A regulators were calculated by Spearman and differential expression analyses. One-way analysis of variance and the Kruskal–Wallis test were utilized to perform comparisons among three groups. Based on the correlation between m6A score and patient survival, the R package of “survminer” was used to determine the cutoff point for each dataset subgroup. Patients were then divided into the high-m6A score group or low-m6A score group based on the maximally selected rank statistics. The survival curves for the prognostic analysis were generated using the Kaplan–Meier method and log-rank test to identify the significance of differences. Univariate and multivariate Cox regression analyses were used to confirm the prognostic value of m6A score and various clinical characteristics. All statistical analyses were performed with R version 3.6.3.

## Results

### The Genetic Variation Landscape of m6A Regulatory Factors in Bladder Cancer

**Figure 1:**
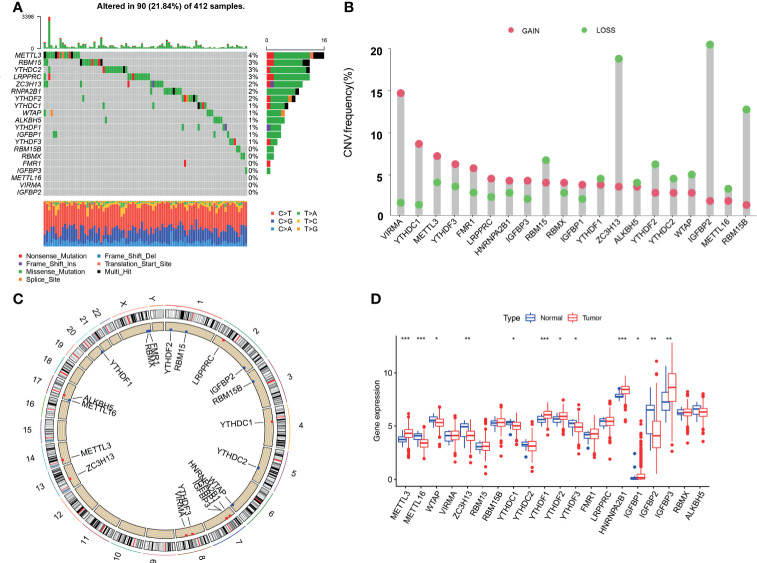
Genetic landscape and expression changes of the m6A regulator in bladder cancer. **(A)** Mutation frequency of the m6A regulators of bladder cancer patients in the TCGA-BLCA cohort. **(B)** A histogram plotting the mutation frequency of each gene obtained by statistical analysis of the copy number of m6A. The abscissa was the m6A-related gene, and the ordinate was the mutation frequency. Red represents an increase in copy number, and green represents loss of copy number. **(C)** The m6A copy number circle graph. Red represents the sample with missing gene copy number than the sample with increasing copy number. Green represents the sample with missing gene copy number than the sample with increasing copy number. **(D)** The box plot of m6A differential expression analysis. Red represents the tumor sample, and green represents the normal sample. The *** represents p < 0.0001, ** represents p < 0.01, * represents p < 0.05.

This study identified 20 m6A regulators in bladder cancer, including 12 readers, 7 writers, and 1 eraser. **Figure 1A** shows the incidence of copy number variation and somatic mutations of the m6A regulatory factors in bladder cancer. **Figure 1B** shows the mutation frequency of each gene obtained by statistical analysis of the copy number of m6A. **Figure 1C** shows the m6A copy number circle diagram, which shows the position of the CNV mutation of the m6A regulatory factor on the chromosome. **Figure 1D** represents a further analysis of the m6A difference. The m6A-related gene difference analysis between normal samples and tumor samples indicated that CNV mutations may be significantly related to m6A modulator expression disorder. Compared with normal tissues, the expression of CNV-increased m6A modulators of bladder cancer tissues (such as METL3 and YTHDF1) was significantly increased. Conversely, the expression of CNV-deficient m6A modulators of bladder cancer tissues (such as ZC3H13 and WTAP) was reduced.

### Identification of m6A Methylation Modification Patterns Mediated by Regulators

**Figure 2:**
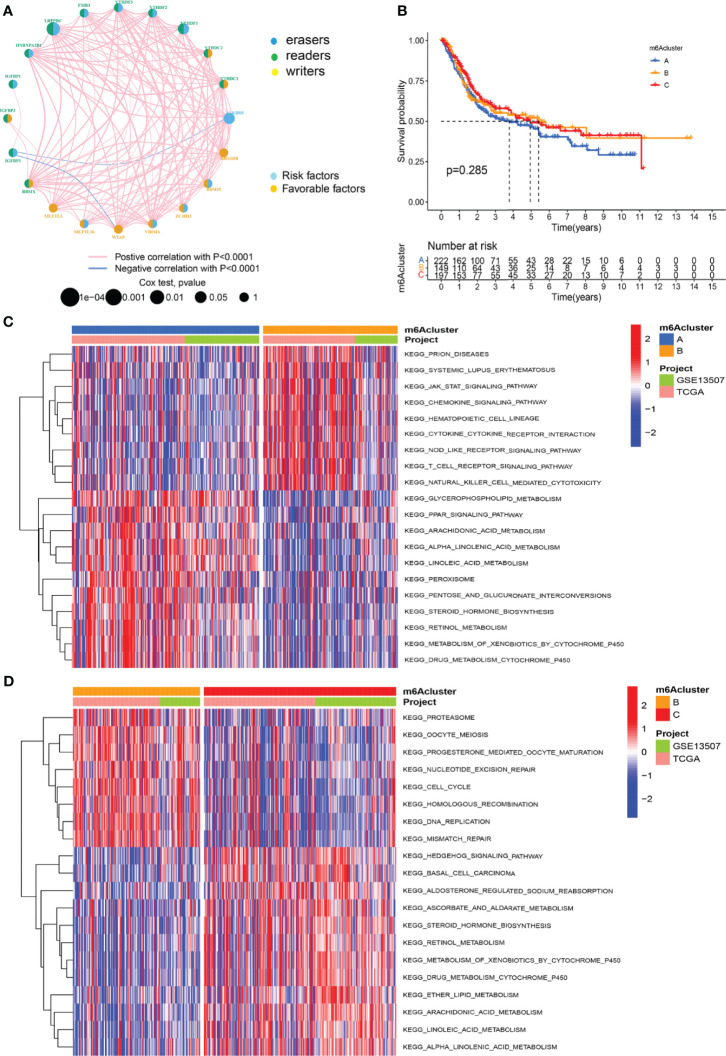
M6A methylation modification patterns and the biological characteristics of each pattern. **(A)** M6A regulates the prognosis network diagram. **(B)** Survival analysis for m6A modification patterns. **(C)** GSVA analyzed the differences between functional pathways in m6A modification patterns. Blue represents the m6A modification pattern A, and orange represents the m6A modification pattern B. **(D)** GSVA analyzed the differences between functional pathways in m6A modification patterns. Orange represents the m6A modification pattern B, and red represents the m6A modification pattern C.

The GSE13507 (N = 165) of the GEO database and TCGA-BLCA (N = 403) datasets with complete survival data and corresponding clinical information were included to match the RNA samples. The m6A prognosis network diagram showed that most of the expression of m6A-related genes were positively correlated, with only negative correlations between IGFBP3 and ALKBH5, IGFBP3, and WTAP (**Figure 2A**). Based on the expression of m6A regulators, three modification patterns were eventually identified (**Figure S1**). The survival analysis of the m6A modification pattern showed that patients with modification patterns B and C had better survival rates than pattern A patients (**Figure 2B**).

### Characteristics of TME Cell Infiltration Under Different m6A Modification Patterns

**Figure 3:**
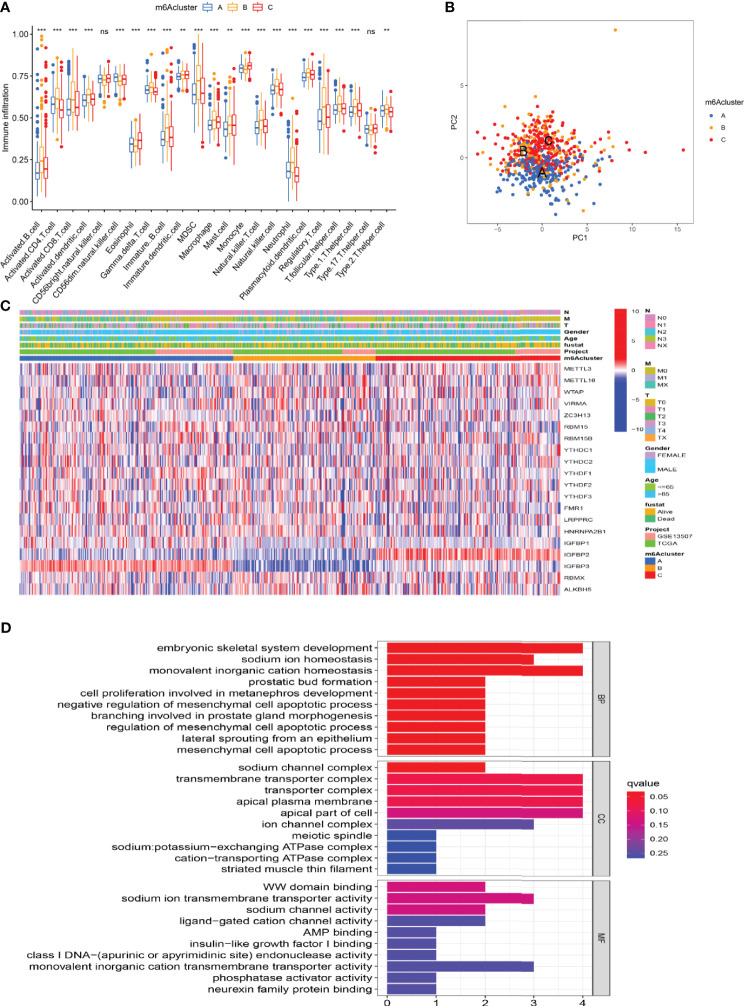
Characterization of TME cell infiltration and transcriptome features in three m6A modification patterns. **(A)** The differential expression analysis of 23 immune cells among three m6A modification patterns. The *** represents p < 0.0001, ** represents p < 0.01 and ns represents no significance. **(B)** The scatter plot of PCA analysis. **(C)** Unsupervised clustering of 20 m6A regulators of bladder cancer. **(D)** GO enrichment analysis of m6A-related genes.

We used GSVA analysis to investigate the differences in biological function of different m6A modification patterns. As shown in **Figure 2C**, we observed the difference in functional pathways between different patterns. M6Acluster-A were mainly concentrated in stromal and carcinogenic activation pathways; m6Acluster-B were associated with immune activation, including the activation of the chemokine signaling pathway, T cell receptor signaling pathway, cytokine–cytokine receptor interaction, and Jak stat signaling pathways; and m6Acluster-C was significantly associated with immune desert biological processes (**Figure 2D**). Subsequent analysis of TME cell infiltration showed that m6Acluster-B was significantly enriched for innate immune infiltration of cells, including macrophages, mast cells, eosinophils, MDSC cells, and plasmacytoid dendritic cells. Three m6A modification patterns showed significantly different infiltration characteristics of TME cells (**Figure 3A**). The results of the PCA analysis showed significant differences between the transcriptome profiles of the three m6A modification patterns (**Figure 3B**). The heat map shows that m6A-related genes were highly expressed in m6Acluster-A, while most genes were negligibly expressed in m6Acluster-B and m6Acluster-C (**Figure 3C**). GO enrichment analysis showed that the differential genes were mainly enriched in the biological process (BP), embryonic skeletal system development, sodium ion homeostasis, and monovalent inorganic cation homeostasis (**Figure 3D**).

### Construction of m6A Gene Signature and Functional Annotation

**Figure 4:**
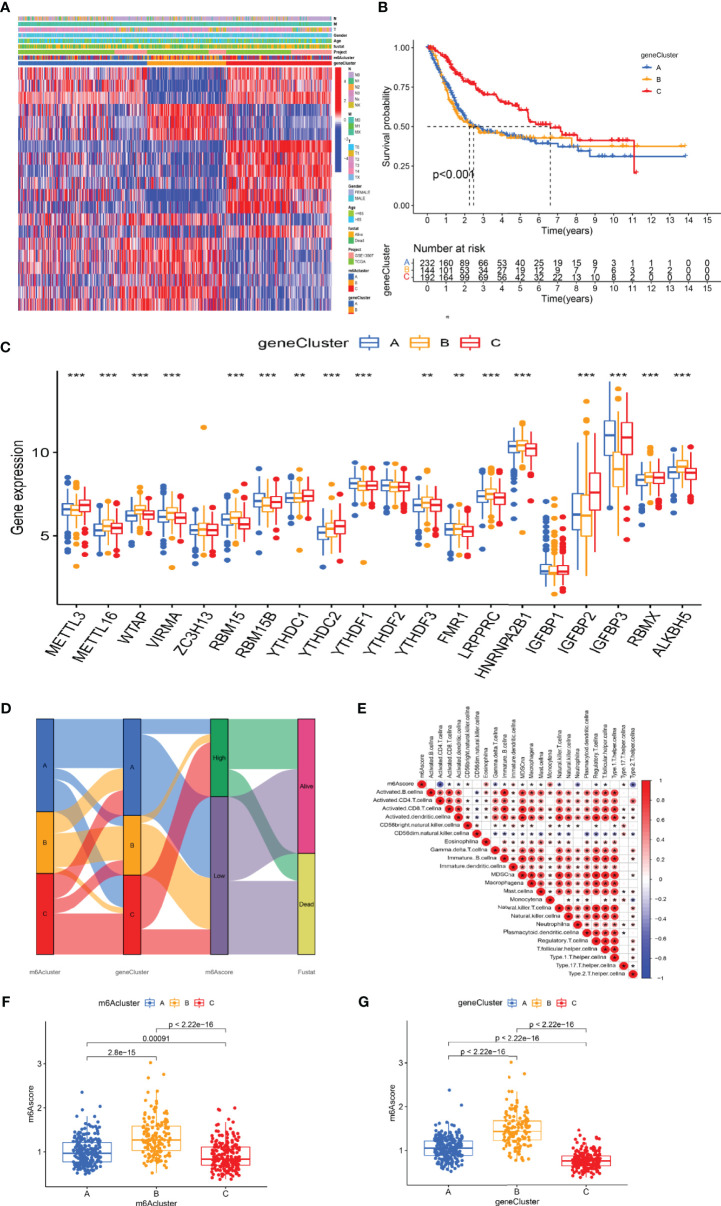
The development of m6A signature. **(A)** The heat map of genetic modification patterns. **(B)** Survival curves of different gene-Clusters. **(C)** Box plot of the differential expression analysis of m6A-related genes among different gene-Clusters. The *** represents p < 0.0001, ** represents p < 0.01. **(D)** Sankey diagrams of different genotypes. **(E)** The correlation analysis between the m6A score and immune cells, with red indicating positive correlation and blue indicating negative correlation. **(F)** Differential expression analysis of the m6A score in the m6A cluster. **(G)** Difference analysis of the m6A score in the gene-Cluster.

In addition, we identified 44 m6A phenotype-associated DEGs. **Table 1** shows that univariate Cox regression analysis identified differential genes related to the prognosis of bladder cancer. Consistent with the m6A modification pattern, the unsupervised clustering algorithm also revealed three m6A modification genomic phenotypes (gene-Cluster A, gene-Cluster B, and gene-Cluster C) (**Figure S2**). The heat map of genetic modification patterns included clinical information. Most genes were low-expressed in gene-Cluster B and high-expressed in gene-Cluster C (**Figure 4A**).

**Table 1:** Univariate Cox regression analysis of differential genes in bladder cancer.

Gene	HR	95% *CI*	*p* value
TNFRSF21	0.9046	0.8186–0.9995	0.0490
SCNN1G	0.9328	0.8773–0.9919	0.0263
KRT7	0.9496	0.9041–0.9974	0.0389
STX2	1.2006	1.0597–1.3602	0.0041
DNAJB5	1.1432	1.0032–1.3026	0.0446
TNFAIP8L3	1.2044	1.0868–1.3348	0.0004
CRTAC1	0.9089	0.8614–0.9589	0.0005
ALDH1L1	0.9112	0.8494–0.9775	0.0094
ATOH8	0.8672	0.7941–0.9471	0.0015
KLHL3	0.7762	0.6638–0.9077	0.0010
ATP1A4	0.8503	0.7612–0.9498	0.0041
SHH	0.8887	0.8166–0.9671	0.0062
RBL1	1.3445	1.1126–1.6248	0.0022
MPPED2	0.8573	0.7570–0.9709	0.0153
KIFC1	1.2270	1.0540–1.4285	0.0083
CDKN3	1.2234	1.0844–1.3802	0.0010
RRM2	1.1268	1.0063–1.2616	0.0385
SBSN	1.0876	1.0370–1.1407	0.0006
NEIL3	1.1924	1.0313–1.3785	0.0175
TPM3	1.2968	1.0539–1.5957	0.0141

HR, hazard rate; CI, confidence interval.

Further survival analysis revealed significant differences among the three m6A modification genomic phenotypes in bladder cancer (*p* < 0.001). The survival curve showed that patients with gene-Cluster C had the worst prognosis (**Figure 4B**). M6A regulators were the source of prominent differences in the three m6A modification genomic phenotypes (**Figure 4C**). We developed an m6 score based on the m6A-related signature to quantify the m6A modification patterns in individual bladder cancer patients. Patients were divided into the high-m6A score group and the low-m6A score group according to the optimal cutoff value (1.3530). The alluvial diagram showed the flow of m6A score fraction construction (**Figure 4D**). Immune correlation analysis showed that the m6A score was significantly positively correlated with CD4 T immune cells, CD8 T immune cells, and dendritic immune cells (**Figure 4E**). The m6A score differed not only in the m6Acluster but also in the gene-Cluster. Differential expression analysis of m6A score in m6Acluster showed the highest score was in m6Acluster-B compared to the other clusters (**Figure 4F**). The highest score was in gene-Cluster B (**Figure 4G**).

### Modification Characteristics of Molecular Subtype m6A and Tumor Somatic Mutations

**Figure 5:**
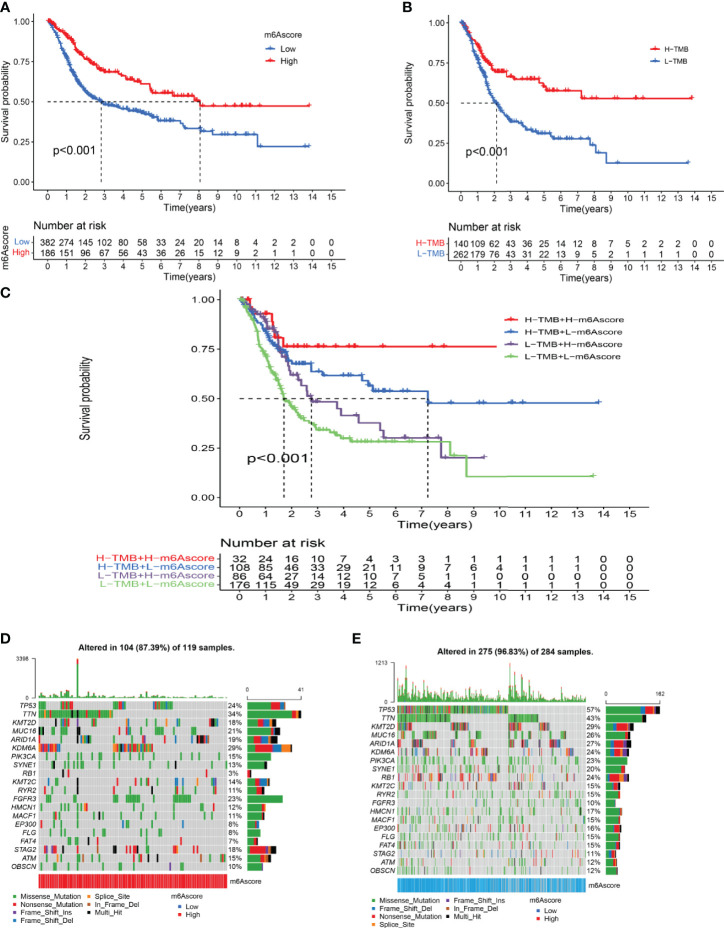
Characterization of m6A modifications in tumor somatic mutation. **(A)** Survival analysis of the high-m6A score group and low-m6A score group. **(B)** Survival analysis of tumor mutation burden. **(C)** Survival analysis of TMB combined with m6A score. **(D)** Waterfall chart of the high-m6A score group. **(E)** Waterfall chart of the low-m6A score group.

Survival analysis showed that the prognosis of patients in the low-m6A score group was poorer than that in the high-m6A score group (*p* < 0.001) (**Figure 5A**). Bladder cancer samples were divided into a high mutation load group and a low mutation load group according to the expression of tumor mutation burden (TMB) (4.6578). Survival analysis of tumor mutation burden revealed that the prognosis of the group with a high tumor mutation burden was better than that of patients with a low tumor mutation burden (*p* < 0.001) (**Figure 5B**). More importantly, the survival curve of TMB combined with the m6A score showed that the patients in both the low tumor mutation group and the low-m6A score group had the worst prognosis (**Figure 5C**). The frequency (96.83%) was higher than the total gene mutation frequency of the high-m6A score group (87.39%) (**Figures 5D, E**).

### M6A Clinical Correlation Analysis

**Figure 6:**
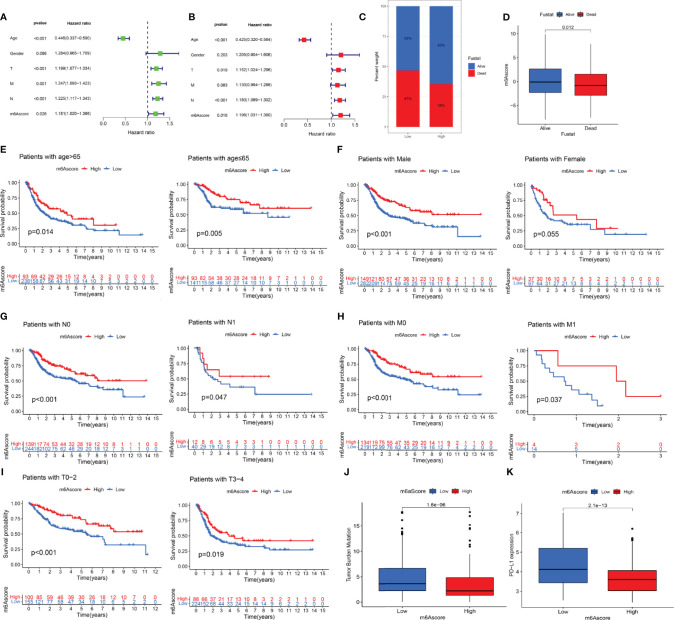
The prognostic value of the m6A score and the correlation between the clinicopathological features and m6A score. **(A)** Univariate Cox regression analysis of the m6 score for bladder cancer was shown by forest plot. **(B)** Multivariate Cox regression analysis of m6 score for bladder cancer was shown by forest plot. **(C)** Stratified analysis of the m6A score for bladder cancer patients by status. **(D)** Stratified analysis of the m6A score for bladder cancer patients by status. **(E)** Stratified analysis of the m6A score for bladder cancer patients by age. **(F)** Stratified analysis of the m6A score for bladder cancer patients by gender. **(G)** Stratified analysis of the m6A score for bladder cancer patients by N. **(H)** Stratified analysis of the m6A score for bladder cancer patients by M. **(I)** Stratified analysis of the m6A score for bladder cancer patients by T. **(J)** Stratified analysis of the m6A score for bladder cancer patients by tumor mutation burden. **(K)** Stratified analysis of the m6A score for bladder cancer patients by PD-L1.

According to the results of univariate and multivariate Cox regression analyses, the m6 score was identified as an independent prognostic variable of bladder cancer (**Figures 6A, B**). Through the survival analysis, we found that bladder cancer patients died mainly in the low-m6A score group (**Figure 6C**). The log-rank test showed that the survival time was significant between the high-m6A score group and the low-m6A score group (**Figure 6D**). Stratified analysis showed that patients in the high-m6A score group had a better prognosis than patients in the low-m6A score group of the male, N0, N1, M0, M1, T0–2, and T3–4 (**Figures 6E–I**). Based on the risk stratification analysis of tumor mutation burden, **Figure 6J** shows that m6A was suitable for the high- and low-score groups of tumor mutation burden (*p* = 1.6e-06). PD-L1 played an important role in bladder cancer. **Figure 6K** shows that PD-L1 made a difference between the high-m6A score group and the low-m6A score group (*p* = 2.1e-13).

### Immunotherapy Analysis

**Figure 7:**
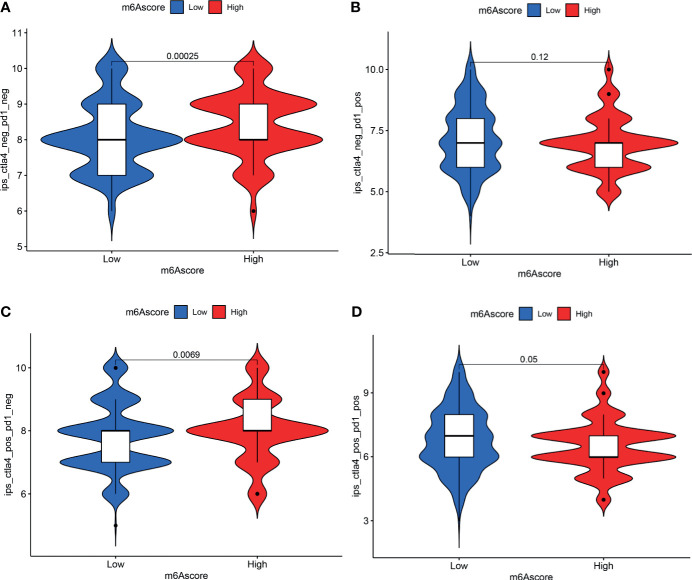
Analysis of m6A modification patterns in anti-PD-L1 and CTLA-4 immunotherapy. **(A)** Differential analysis for low-m6A score group and high-m6A score group in CTLA-4 negative and PD-L1 negative therapy. **(B)** Differential analysis for low-m6A score group and high-m6A score group in anti-PD-L1 immunotherapy. **(C)** Differential analysis for low-m6A score group and high-m6A score group in anti-CTLA-4 immunotherapy. **(D)** Differential analysis for low-m6A score group and high-m6A score group in anti-PD-L1 combined with CTLA-4 immunotherapy.

Analysis of immunotherapy scores in the high-m6A score and low-m6A score groups showed that ICI therapy represented by the CTLA-4/PD-1 inhibitor played an important role in antitumor therapy. **Figure 7A** shows CTLA4 negative and PD-L1 negative therapy was different between the high-m6A score group and low-m6A score group (*p* = 0.00025). PD-1 immunotherapy showed no difference between the high-m6A score group and low-m6A score group (**Figure 7B**). **Figure 7C** shows CTLA-4 immunotherapy was different between the high-m6A score group and the low-m6A score group (p = 0.0069). CTLA-4/PD-1 immunotherapy showed no difference between the high-m6A score group and low-m6A score group (**Figure 7D**).

## Discussion

Determining the role of m6A RNA methylation modification in tumor mutation burden cell infiltration will help understand the mechanism of TME antitumor immune response. In this study, we confirmed three m6A methylation modification patterns based on 20 m6A regulators characterized by different immune phenotypes. The combination of TME cell infiltration characteristics in different m6A modification patterns will improve the knowledge of TME antitumor immune response of bladder cancer.

In this study, we found that three m6A methylation modification patterns had a significant correlation with immune activation and other pathways. M6Acluster-A was characterized by the activation of immunity and lymphocyte infiltration. M6Acluster-B featured the presence of immune cells, as well as the activation of EMT and Wnt signaling pathways, which was consistent with the immune rejection phenotype. M6Acluster-C was consistent with the immune desert phenotype. The immune rejection phenotype showed the presence of a large number of immune cells and the forming of immune cells inside the cancer (8). The immune desert phenotype was related to immune tolerance and lack of activated and initiated T cells (24). The above studies were in line with our findings. This confirmed that m6A modification patterns had a very important significance in shaping a different TME landscape. Many recent studies have found that the biological functions of immune cells play an important role in the TME and cancer immunotherapy (25, 26). The relevant immune cells in the TME mainly included antitumor immune cells and tumor-promoting immune cells. It is worth noting that these two types of cells play different roles in different stages of tumor progression. Antitumor immune cells mainly include effector T cells (CD8+ cytotoxic T cells and effector CD4+ T cells) and dendritic cells (27). The mechanism of CD4+ T cells was to use the cross to provide tumor antigens and costimulatory molecules to CD8+ T cells, allowing dendritic cells to activate CD8+ T cells (28, 29); hence, a comprehensive analysis of the m6Acluster will help us understand the infiltration characteristics of TME cells.

Further, reflecting the results for m6A modification patterns, m6A-related signature genes’ differences were related to the immune-related pathway. This demonstrated the importance of m6A modification patterns in shaping variant TME landscapes. Due to the heterogeneity and specificity of m6A-modified individuals, we constructed a score model to assess the m6A modification pattern of individual patients with bladder cancer. The m6A modification pattern of the immune rejection phenotype had a higher m6A score, while the m6A modification pattern of the immunoinflammatory phenotype had a lower m6A score. The m6A score was positively correlated with CD4 T immune cells, CD8 T immune cells, and dendritic immune cells. This indicated that the m6A score was a dependable and stable tool for the comprehensive assessment of the modification pattern of individual tumor m6A. In addition, while univariate and multivariate Cox regression analysis indicated that the m6A score may be an independent prognostic factor, a study has suggested distinguishing between invasive and non-invasive micropapillary carcinoma of the bladder, as the latter may not predict a poor prognosis (30). Variant histology may be related to survival outcomes (31). Further studies on the relationship between variant histology and m6A are still needed. Even so, we observed that the m6A score was strongly related to the tumor immunophenotype. The frequency of gene mutations in the low-m6A score group was higher than the total gene mutation frequency in the high-m6A score group. The immunotherapy scores of the high-m6A score and low-m6A score group were different. There are different treatment methods for CTLA-4 immunotherapy between the high and low groups. The high-m6A score group of bladder cancer patients had obvious clinical advantages. This indicated that m6A modification may influence the curative effect of immunotherapy.

Previous studies had shown that m6A-related genes, including METTL3, were negatively correlated with the recurrence of bladder cancer patients (32, 33). The expression of the catalytic subunit METTL3 of MTC was significantly upregulated in bladder cancer tissues and was related to the development and progression of bladder cancer patients (25). Studies also found that YTHDF1/YTHDF3 can preferentially identify the m6A-modified region in the 3 untranslated regions of ITGA6, promoting ITGA6 translation and enhancing the growth and metastasis of bladder cancer cells (33, 34). ALKBH5 can demethylate CDCP1 and regulate CDCP1 protein expression negatively (35). The expression level of METTL14 of bladder cancer and tumor-initiating cells showed a decrease, and it was significantly related to the clinical severity and prognosis of bladder cancer (36). The molecular mechanism and cellular effect of m6A RNA methylation modification of other molecules, especially methylation recognition proteins, were not fully understood in bladder cancer, with different or the same methyltransferases or demethylases working in different ways. The evaluation of mutational driver genes based on tumor was the key basis for cancer diagnosis and treatment. The results showed that, compared with the high-m6A score group, the mutation rate of TP53 in the low-m6A score group was significantly higher, while the TTN mutation rate in the high group was increased. Previous studies had shown that different TP53 mutations found in separate clusters of tumor may also cause TP53 mutations at a later stage. Detection of TP53 mutations can help identify early-stage lesions that are at high risk of development (37). TTN mutations in tumors will increase, while its immunostimulatory characteristics will also appear higher. At the same time, it has been found that the TTN mutation load represents a high TMB state (38). This indicates intricate interactions between different modifications of m6A and immune genes in the TME. The abnormal expression mechanism of m6A RNA methylation modification regulatory molecules in bladder cancer is still unclear, so we need to develop a new treatment method based on m6A RNA methylation modification to regulate the TME.

In its clinical and practical applications, our study has its advantages. First, the m6A score may be used to assess m6A methylation patterns and corresponding TME cell infiltration characteristics in individual bladder cancer patients to further define the immune phenotype of tumor. Second, after investigating the association between m6A score and clinicopathological features, we suggest that the m6A score may be used as an independent prognostic biomarker for patients with bladder cancer. Finally, the m6A score may predict the efficacy of CTLA-4 immunotherapy in patients with bladder cancer, providing new insights that may guide individualized treatment of patients with bladder cancer. The current study has a few limitations that need to be acknowledged. On the one hand, we only explored the molecular mechanism of m6A modification through 20 RNA methylation regulatory factors that had been identified, while no other regulatory factors were incorporated into the m6A modification mode. On the other hand, we did not explore the relationship between m6A modification and the variant histology of bladder cancer. We therefore need to introduce new regulatory factors and clinicopathological features to improve the accuracy of the model in future studies.

## Conclusions

In this study, we comprehensively assessed the m6A modification patterns based on 20 m6A regulators. The difference in m6A modification patterns may be an important factor in the diversity and complexity of individual TME. The assessment of m6A modification patterns in individual bladder cancer will enhance our knowledge of TME infiltration characteristics and provide the basis for guiding immunotherapy strategies.

## Data Availability Statement

The datasets presented in this study can be found in online repositories. The names of the repository/repositories and accession number(s) can be found in the article/**Supplementary Material**.

## Author Contributions

HZ designed this study. HZ, XS, YY, XJ and ZS downloaded and analyzed the data. HZ wrote this manuscript. HZ, YY, NW and YW explained the data. YY reviewed and revised the manuscript. All authors contributed to the article and approved the submitted version.

## Funding

This research was funded by the National Natural Science Foundation of China (No. 82073670).

## Conflict of Interest

The authors declare that the research was conducted in the absence of any commercial or financial relationships that could be construed as a potential conflict of interest.

## Publisher’s Note

All claims expressed in this article are solely those of the authors and do not necessarily represent those of their affiliated organizations, or those of the publisher, the editors and the reviewers. Any product that may be evaluated in this article, or claim that may be made by its manufacturer, is not guaranteed or endorsed by the publisher.
