# Supplementary figures and images for: M6A Classification Combined With Tumor Microenvironment Immune Characteristics Analysis of Bladder Cancer
Source: Front Oncol. 2021 Sep 15;11:714267. doi: 10.3389/fonc.2021.714267 (PMC8479184; doi:10.3389/fonc.2021.714267)

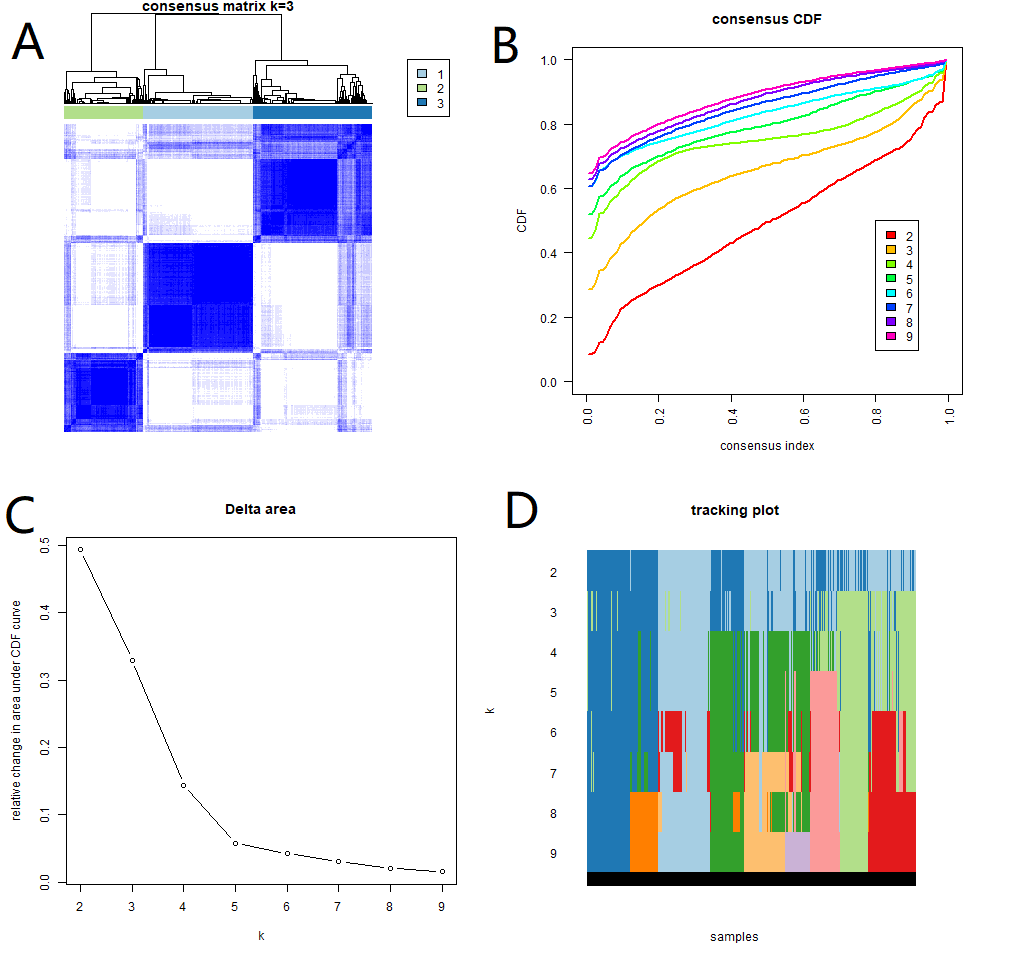

Supplement: Supplementary file 1 [file Image_1.tif]

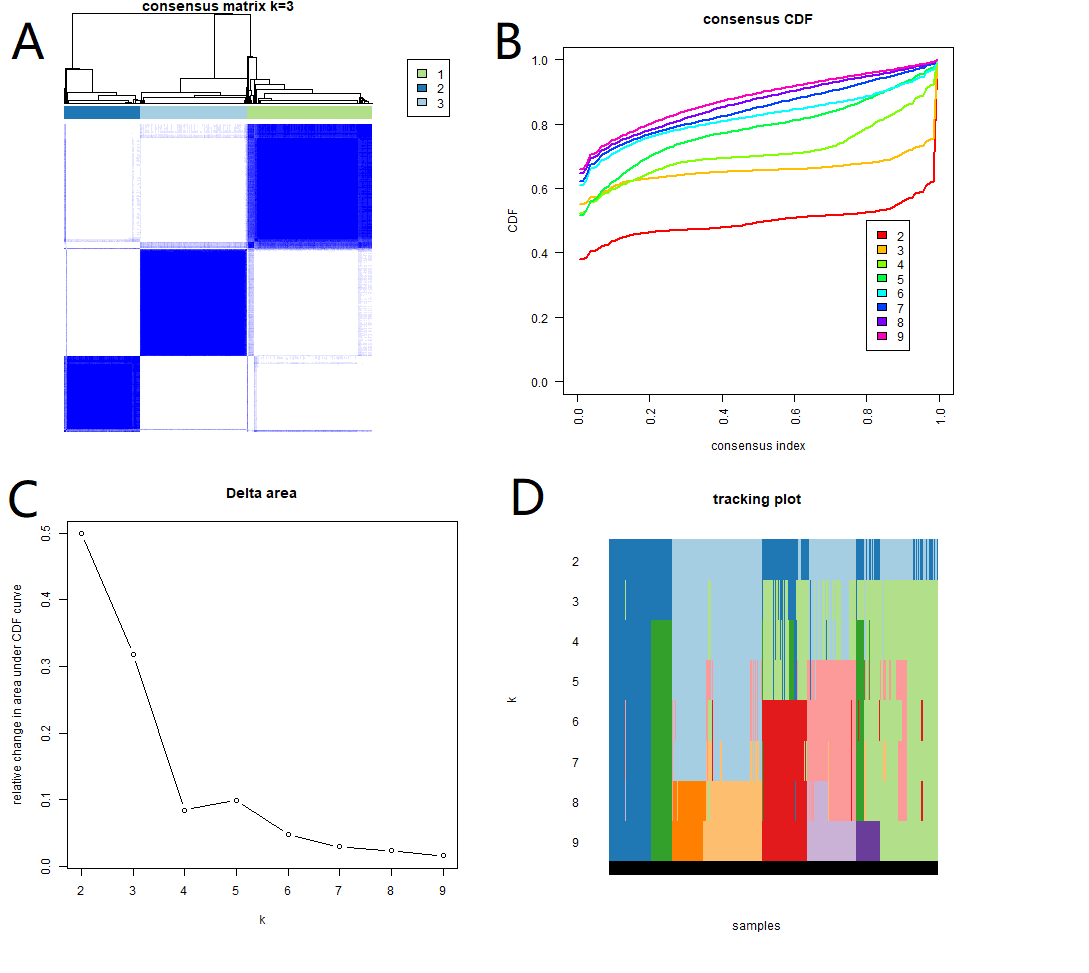

Supplement: Supplementary file 2 [file Image_2.tif]
